# Potential role between inflammatory cytokines and Tie-2 receptor levels and clinical symptoms in patients with first-episode schizophrenia

**DOI:** 10.1186/s12888-023-04913-7

**Published:** 2023-07-25

**Authors:** Fanfan Yan, Xiaojing Meng, Xialong Cheng, Wenzhi Pei, Yuanyuan Chen, Long Chen, Mingming Zheng, Li Shi, Cuizhen Zhu, Xulai Zhang

**Affiliations:** 1 Affiliated Psychological Hospital of Anhui Medical University, Hefei, 230022 China; 2 Anhui Clinical Center for Mental and Psychological Diseases, Hefei, 230022 China; 3 Hefei Fourth People’s Hospital, Hefei, 230022 China; 4 Anhui Mental Health Center, Hefei, 230022 China

**Keywords:** Cardiovascular, First-episode schizophrenia, Inflammatory cytokines, Tie-2

## Abstract

**Background:**

Schizophrenia (SCZ) is associated with chronic low-grade inflammation, which may be involved in the underlying pathological mechanism of the disease and may influence patient prognosis. We evaluated the differences in serum cytokine and Tie-2 receptor levels between patients with first-episode SCZ and healthy controls and explored the correlation thereof with clinical symptoms.

**Methods:**

Seventy-six participants were recruited for the present study, including 40 patients with first-episode SCZ and 36 healthy controls. Positive and Negative Syndrome Scale (PANSS) and Brief Psychiatric Rating Scale (BPRS) scores, demographic data, and blood samples were collected at baseline. A hypersensitive Meso Scale Discovery (MSD) electrochemiluminescence assay system was used to measure cytokine and Tie-2 receptor levels. Spearman’s correlation and stepwise linear regression were used to analyze the data.

**Results:**

Serum interleukin-1β and -4 levels were significantly increased, and Tie-2 levels were significantly decreased, in first-episode SCZ patients as compared to healthy controls. IL-1β levels were positively correlated with total BPRS scores, resistance subscores, and PANSS positive subscores. Furthermore, IL-1β levels were negatively correlated with Tie-2 receptor expression levels. Stepwise linear regression analysis demonstrated that IL-1β levels correlated positively with PANSS positive subscores and BPRS total scores. PANSS negative subscores, general psychopathology subscores, and PANSS total scores had positive effects on the Tie-2 receptor. Receiver operating characteristic (ROC) curve analysis showed that IL-1β and Tie-2 were highly sensitive and specific for predicting first-episode SCZ symptoms and achieving an area under the ROC curve of 0.8361 and 0.6462, respectively.

**Conclusion:**

Our results showed that patients with first-episode SCZ have low-grade inflammation. IL-1β and Tie-2 receptors may be important mediators between inflammation and vascular dysfunction in patients with SCZ and may underlie the increased cardiovascular disease in this population.

**Trial registration:**

The clinical trial registration date was 06/11/2018, registration number was chiCTR1800019343.

## Background

Schizophrenia (SCZ) is a common, severe mental disorder of unknown etiology that affects approximately 1% of the world’s population [1, 2]. It usually begins in early adolescence and is characterized by disorganized thinking, lack of motivation, emotional apathy, and cognitive impairment [3]. Previous studies have identified SCZ as a neurodevelopmental disorder caused by pro-inflammatory risk genes, environmental stress factors, and immune system alterations [4, 5]. The vulnerability–stress–inflammation hypothesis could help explain the key role of inflammation in SCZ. Stress may increase the expression of pro-inflammatory cytokines and thereby contribute to the development of SCZ symptoms [6, 7]. However, current evidence on the relationship between inflammation and SCZ pathogenesis is heterogeneous and uncertain, which could imply that the present literature has inadequately accounted for the effect of confounding factors on the mechanisms underlying SCZ. Thus, further novel evidence is needed to identify the link between the immune system and SCZ and to provide useful suggestions for future research or improvements. Indeed, resurgent interest in this research area has arisen in recent years.

Inflammation is the first step in the immune response [8]. Well-regulated inflammatory processes are crucial for the homeostasis and normal function of tissues. Excessive inflammatory responses can lead to host cell damage [9]. Cytokines are key molecules in the regulation of inflammation and are important mediators between the brain and the immune system. Cytokines can affect neurodevelopmental, neuroendocrine, and neurotransmission processes through inflammatory immune responses, leading to structural or functional brain damage and thereby contributing to SCZ psychopathology [10]. Increasing evidence has demonstrated that cytokines, such as interleukin (IL)-1, IL-2, IL-4, IL-6, IL-8, IL-10, and tumor necrosis factor (TNF)-α, cause changes in the peripheral blood of patients with SCZ [10, 11]. Numerous studies have shown that the use of anti-inflammatory drugs as adjuncts to antipsychotics is superior to a placebo in SCZ patients [12], providing evidence for immune-based therapy for symptom improvement. However, inconsistent conclusions have been drawn regarding the effects of abnormal expression of inflammatory cytokines on SCZ symptoms due to dissimilarities in the clinical SCZ status of patients.

In addition, inflammation is associated with increased vascular risk and contributes to the subsequent development of hypertension, atherosclerosis, and ischemic stroke [13–15]. Notably, cardiovascular disease risk is significantly higher in patients with SCZ than in the general population [16, 17]. Maladaptive and persistent inflammatory responses may be linked to the development and maintenance of cardiovascular diseases in SCZ [18]. Endothelial tyrosine kinase receptors with immunoglobulin and epidermal growth factor homology domain 2 (Tie-2) play a key role in inflammation-related pathological angiogenesis and inflammatory processes [19]. The function of Tie-2 and inflammatory cytokines, such as IL-1β, IL-6, and TNF-α, in vascular inflammation has been verified in tumor angiogenesis, retinal angiogenesis, arthritis, and peripheral artery diseases [19–23]. However, to date, no study has simultaneously reported the levels of Tie-2 and inflammatory cytokines in patients with first-episode SCZ.

In this study, we investigated whether serum inflammatory cytokines and Tie-2 receptor levels were altered in first-episode SCZ patients and explored the relationship between cytokines and Tie-2 receptor levels and psychopathological symptoms, to provide new feasible ideas for clinical intervention and assessing prognosis in SCZ.

## Methods

### Participants

Participants with first-episode SCZ were recruited from the patients attending the Hefei Fourth People's Hospital between January 2019 and December 2020. A total of 40 patients met the following inclusion criteria: (1) fulfillment of the Diagnostic and Statistical Manual of Mental Disorders, Fifth Edition (DSM-5) criteria for first-episode state and drug-naïve SCZ or taking antipsychotic drugs for < 1 week, as diagnosed by two independent, experienced psychiatrists; (2) no acute infectious disease or trauma in the past 1 month and no corticosteroid use; (3) aged between 18 and 65 years. The exclusion criteria were as follows: (1) a history of craniocerebral trauma, organic cerebral diseases, or other mental disorders, such as bipolar disorder and major depressive disorder; (2) a history of alcohol or other substance abuse; (3) pregnant or lactating women; (4) diabetes, hypertension, or metabolic or endocrine diseases. The majority of patients were antipsychotic drug-naïve at the time of blood sampling. Patients were given anxiolytics in low doses when needed. Six patients were medicated on the day of recruitment (chlorpromazine equivalent dosage: 150 ± 72.3 mg/day, treatment duration: 2.8 ± 1.5 days). During the same period, healthy controls with no history of mental illness were recruited. All these participants confirmed that they had no major diseases, such as diabetes, renal failure, liver disease, inflammatory diseases, celiac disease, lactose intolerance, or immunodeficiency, in the past or present and had not undergone any abdominal surgery that might have affected oxidative stress and inflammatory marker levels. In addition, no immunosuppressants or anti-inflammatory drugs had been administered to these individuals within the previous 3 months.

### Clinical assessments

#### Mini-international neuropsychiatric interview 6.0.0

Participants were screened for inclusion in this study by two experienced psychiatrists, and the preliminary clinical diagnosis was validated using the Mini-International Neuropsychiatric Interview (MINI) 6.0.0, which is a brief diagnostic interview for mental disorders used by psychiatrists in the United States and Europe. All patients underwent the MINI 6.0.0 evaluation to confirm the clinical diagnosis of the first psychotic state [24].

### Demographic characteristics

The sociodemographic data of the patients, such as sex, age, and body mass index (BMI), were collected from the inpatient electronic medical record system. Face-to-face interviews were conducted by trained health workers to collect demographic and clinical information on healthy populations, including the following information: age, sex (“male” or “female”), height, and weight. BMI was calculated and classified according to the World Health Organization criteria [25].

### Positive and negative syndrome scale

The Positive and Negative Syndrome Scale (PANSS) is widely used to measure severe psychopathology in adult patients with SCZ. The PANSS consists of 30 items divided into three separate subscales, with scores ranging from 30 to 210 points. The positive, negative, and general psychopathology subscales are normally distributed and independent of each other, and the score for each item gradually increases according to the severity of psychiatric symptoms, ranging from 1 to 7 points. Higher PANSS and subscale scores indicate more severe psychiatric symptoms [26].

### Brief psychiatric rating scale

The Brief Psychiatric Rating Scale (BPRS) is an 18-item rating scale that includes five subscales: affective positive symptoms, negative symptoms, resistance, and activation. A higher total score reflects increased disease severity. The factor scores reflect the clinical characteristics of the disease. The total score indicates the overall level of mental symptoms and is often used to evaluate psychopathological changes in patients with SCZ [27].

### Laboratory evaluation

Venous blood samples were collected from all participants between 7 a.m. and 8 a.m. the day after an overnight fast. Venous blood samples (approximately 5 mL) were collected by duty nurses. Each sample was stored at room temperature for 30 min, sent to the laboratory, and centrifuged at 3,000 RPM for 5 min. Before analysis, the serum samples were stored and frozen at − 80 °C. The MSD Platform (Labservice.univ-bio.com, Shanghai, China) was used to measure multiplex levels of inflammatory biomarkers (Tie-2, TNF-α, IL-1β, IL-4, IL-6, and IL-10). MSD sensitivity reaches 0.05 pg/mL, and the effective linear range can reach 6 log, while the conventional linear range of the traditional enzyme-linked immunosorbent assay (ELISA) kit method can only reach 10–1,000 pg/mL.

Biological experiments should evaluate samples from the normal control group and the disease group. The concentration distribution of the protein to be tested in the samples generally ranges from a few tenths of a picogram to several thousand picograms. The linear range of ELISAs cannot simultaneously detect high and low protein abundance. Many pre-experiments are required to explore the appropriate sample dilution, which is time-consuming and laborious. MSD provides a wide linear range of concentrations, from sub-picograms to tens of thousands of picograms, effectively placing all samples within the optimal linear range and achieving accurate determination [28]. For current research projects in which the concentration of the protein to be detected is downregulated in some diseases, the MSD detection method can simultaneously detect high and low abundances of proteins. Therefore, it has a greater clinical application value [29].

### Statistical analysis

Statistical analysis was performed using SPSS software (version 16.0; SPSS Inc., Chicago, IL, USA). The normality of the data distribution was tested. Values conforming to a non-normal distribution are expressed as the median and interquartile range (IQR) (25% and 75% percentiles). Chi-square tests were applied to categorical data. Student’s t-tests were applied for continuous data with a normal distribution. For data with a non-normal distribution, the Mann–Whitney U-test was used, and missing values were replaced by mean values. When multiple comparisons exist, R software (V3.6.1) and online versions (https://www.bioincloud.tech) was adopted the false discovery rate (FDR) method to adjust the *p*-value. Spearman’s test was used to examine the correlation between baseline serum cytokine levels and PANSS and BPRS scores. When the severity of SCZ (PANSS and BPRS scores) was analyzed by stepwise linear regression, IL-1β, IL-4, and Tie-2 levels were used as predictors. We performed 10 regressions. For each regression analysis, regression models with backward steps were selected. The area under the receiver operating characteristic (ROC) curve (AUC) was used to assess the clinical translational value of inflammatory factors. All tests were two-tailed, and differences were considered statistically significant at *p* < 0.05.

## Results

### Demographic and clinical details of study participants

The demographic and clinical characteristics of the two groups are presented in Table 1. Overall, 76 participants were included in this study, including 40 patients with first-episode SCZ (28 females and 12 males) and 36 healthy controls (22 females and 14 males). No statistically significant differences were found among the groups in terms of sex, age, and BMI. All patients underwent strict PANSS and BPRS assessments performed by experienced psychiatrists. The total and subscale PANSS and BPRS scores in first-episode SCZ patients are shown in Table 1.

**Table 1:** Comparison of demographic and clinical data between the two groups

Variables	SCZ (*n* = 40)	Controls (*n* = 36)	Z	p
Age(years)	29.5 (23 ~ 46.5)	26.5 (24 ~ 31)	-1.261	0.207
Gender, F,n%	28(70.0)	22(61.1)	-0.81	0.418
BMI(kg/m^2^)	22.8 (20.5 ~ 24.4)	21.6(20.3 ~ 24.1)	-0.962	0.336
PANSS				
Positive	22.3 (5.7)	─	─	─
Negative	21.9 (4.7)	─	─	─
General	45.4 (5.3)	─	─	─
Total	89.6 (10.8)	─	─	─
BPRS				
Affect	6.1 (2.1)	─	─	─
Positive	11.5 (2.8)	─	─	─
Negative	8.3 (2.4)	─	─	─
Resistance	9.2 (3.4)	─	─	─
Activation	6.5 (2.4)	─	─	─
Total	50.1 (10.2)	─	─	─

Note: *BMI* Body mass index, *BPRS* Brief Psychiatric Rating Scale, *PANSS* Positive and Negative Syndrome Scale, *SCZ* schizophrenia

### Inflammatory cytokine concentrations

MSD was used to measure inflammatory cytokine concentrations in patients and healthy control individuals. As presented in Table 2, the serum levels of IL-1β and IL-4 were significantly higher in the case group than in healthy controls (*p* < 0.05), whereas the Tie-2 levels were lower in the patients than in the healthy controls (*p* = 0.029). Moreover, IL-6, IL-10, or TNF-α levels did not differ significantly between the two groups (*p* > 0.05). These results demonstrated that a higher level of inflammation was present in first-episode SCZ patients than in healthy individuals.

**Table 2:** Baseline serum inflammatory cytokine concentrations in both groups

Variables	SCZ	Controls	Z	p
	(*n* = 40)	(*n* = 36)		
IL-1β(pg/ml)	1.391(1.391 ~ 1.391)	0.972(0.972 ~ 0.972)	-5.460	0.000
IL-4(pg/ml)	11.995(0.157 ~ 11.995)	0.015(0.007 ~ 0.015)	-5.890	0.000
IL-6(pg/ml)	0.854(0.651 ~ 1.232)	0.686(0.397 ~ 1.094)	-1.831	0.067
IL-10(pg/ml)	0.311(0.216 ~ 0.684)	0.292(0.199 ~ 0.431)	-0.640	0.522
TNF-α(pg/ml)	1.202(0.952 ~ 1.462)	4.359(0.765 ~ 5.756)	-1.326	0.185
Tie-2(pg/ml)	1991.785(1707.064 ~ 2214.799)	2200.943(1903.859 ~ 2577.607)	-2.190	0.029

Note: *IL-1β* Interleukin-1β, *IL-4* Interleukin-4, *IL-6* Interleukin-6, *IL-10* Interleukin-10, *TNF-α* Tumor necrosis factor-α, *Tie-2* Tyrosine kinase receptor with immunoglobulin and epidermal growth factor homology domain 2, *SCZ* schizophrenia

### Correlations of cytokine levels with psychotic symptom severity

Correlation analyses were performed to demonstrate the relationship between inflammation level and clinical symptom severity in patients with first-episode SCZ. Table 3 shows that the IL-1β level was positively correlated with the BPRS total scores, resistance subscores, and PANSS positive subscores (*p* < 0.05). After correction for multiple comparisons, we found that the IL-1β and BPRS total scores remained significant (*p* = 0.027). This suggests that, as the concentration of IL-1β increased, the clinical symptoms in the case group worsened. In addition, IL-4 and Tie-2 were not associated with symptom severity (*p* > 0.05). Notably, IL-1β levels correlated negatively with Tie-2 receptor expression (*p* < 0.05).

**Table 3:** Correlation between inflammatory cytokine concentrations and clinical symptoms in case group

a, Correlation between inflammatory cytokines and PANSS scores and subscales in patients																					
	Tie-2		IL-1β	IL-4	Positive		Negative		General	Total											
	r		P. adj	r	P. adj		r		P. adj	r		P. adj		r		P. adj	r			P. adj	r
Tie-2	1.000																				
IL-1β	-0.326^*^		-	1.000																	
IL-4	-0.292		-	0.183	-		1.000														
Positive	0.026		0.873	0.373^*^	0.075		-0.006		0.971	1.000											
Negative	0.040		0.809	0.045	0.784		-0.078		0.633	-0.025		-		1.000							
General	0.097		0.550	-0.010	0.953		0.007		0.968	0.220		-		0.417^**^		-	1.000				
Total	0.069		0.971	0.278	0.082		-0.057		0.971	0.574^**^		-		0.615^**^		-	0.754^**^			-	1.000
b, Correlation between inflammatory cytokines and BPRS scores and subscales in patients																					
	Tie-2		IL-1β		IL-4		Affect			Positive	Negative			Resistance			Activation				Total
	r	P. adj	R	P. adj	r	P. adj	r	P. adj		r	P. adj		r	P. adj	r		P. adj	r	P. adj		r
Tie-2	1.000																				
IL-1β	-.326^*^	-	1.000																		
IL-4	-0.292	-	0.183	-	1.000																
Affect	-0.201	0.307	0.142	0.475	-0.146	0.473	1.000														
Positive	-0.224	0.246	0.300	0.136	-0.012	0.943	0.397^*^	-		1.000											
Negative	-0.074	0.707	0.279	0.153	0.095	0.651	0.169	-		0.248	-		1.000								
Resistance	-0.102	0.635	0.365^*^	0.061	-0.089	0.656	0.286	-		0.519^**^	-		0.421^**^	-	1.000						
Activation	-0.258	0.186	0.226	0.246	-0.033	0.871	0.576^**^	-		0.263	-		0.314^*^	-	0.565^**^		-	1.000			
Total	-0.300	0.136	0.417^**^	0.027	-0.032	0.871	0.560^**^	-		0.714^**^	-		0.581^**^	-	0.840^**^		-	0.739^**^	-		1.000

Note: *Tie-2* Tyrosine kinase receptor with immunoglobulin and epidermal growth factor homology domain 2, *IL-1β* Interleukin-1β, *IL-4* Interleukin-4

^*^p ≤ 0.05

^**^p ≤ 0.001, P.adj: adjusted *P*-values

### Factors predictive of clinical symptoms

**Fig. 1:**
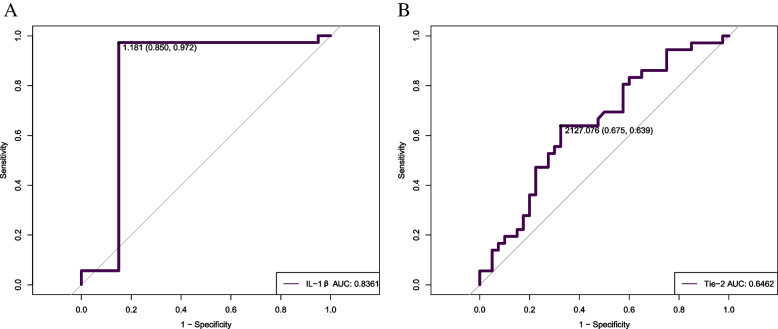
ROC curve analysis of IL-1β and Tie-2 between first-episode schizophrenia patients and healthy controls **A**-**B**. AUC: Area under ROC

Stepwise regression analysis (Table 4) showed that serum cytokine levels can be used as predictors of clinical symptoms. We performed 10 regressions. For each regression analysis, the backward selection method was used for regression models. According to regression analysis, higher IL-1β levels were associated with higher PANSS positive subscores and BPRS total scores. Additionally, a lower Tie-2 level correlated positively with PANSS negative subscores, PANSS general psychopathology subscores, and PANSS total scores. Using ROC curves, we further evaluated the validity and effectiveness of IL-1β and Tie-2 levels for predicting clinical symptom progression in SCZ patients. We found that IL-1β can predict the progression of SCZ at a cutoff level of 1.181 pg/L, with a specificity of 0.850 and sensitivity of 0.972 (Fig. 1A). Tie-2 could predict the progression of SCZ at a cutoff level of 2127.076 pg/L, with a specificity of 0.675 and sensitivity of 0.639 (Fig. 1B). The AUCs of IL-1β and Tie-2 were 0.8361 and 0.6462, respectively. These results suggested that IL-1β and Tie-2 are important predictors of clinical symptom progression in first-episode SCZ.

**Table 4:** Stepwise regression analyses for predictors of clinical symptom

Dependent variables	Independent variables	*B*	*SE*	*Beta(β)*	*T*	*P*	*R*^*2*^
PANSS							
Positive	IL-1β	2.678	1.037	0.386	2.581	0.014*	0.149
Negative	Tie-2	0.003	0.001	0.319	2.183	0.036*	0.370
	IL-4	-0.049	0.028	-0.251	-1.79	0.082	0.278
General	Tie-2	0.003	0.001	0.365	2.297	0.028*	0.293
Total	Tie-2	0.007	0.003	0.376	2.292	0.028*	0.246
BPRS							
Positive	IL-1β	0.936	0.522	0.269	1.796	0.081	0.206
Total	IL-1β	3.826	1.840	0.306	2.079	0.045*	0.237

Note: *Tie-2* Tyrosine kinase with immunoglobulin-like and epidermal growth factor homology domains 2, *IL-1β* Interleukin-1β, *IL-4* Interleukin-4, *PANSS* Positive and negative syndrome, *BPRS* Brief Psychiatric rating scale

^*^p ≤ 0.05

## Discussion

We found that IL-1β and IL-4 levels were significantly elevated in first-episode psychosis in patients diagnosed with SCZ. This result was consistent with some previous studies [30–33], but not with those of others [34–37]. We did not find statistically significant differences in IL-6, IL-10, and TNF-α levels between the case group and healthy controls, similar to some published studies [30, 35, 38]. However, several studies, including meta-analyses, found elevated levels of IL-6, IL-10, and TNF-α in first-episode drug-naïve (FEDN) psychosis patients and first-episode SCZ patients, the majority of whom were using antipsychotics [30–32, 37]. Conversely, Goldsmith, as well as Xiu et al., found reduced levels of IL-10 in first-episode SCZ patients and FEDN patients [30, 39], while another study showed that TNF-α levels were reduced in FEDN patients [40].

Furthermore, the BPRS total scores, resistance subscores, and PANSS positive subscores were positively correlated with IL-1β levels. These findings were consistent with those of a previous study [32]. Further stepwise linear regression analysis also showed that IL-1β levels were significantly associated with positive symptoms and the total scale score. Our results suggested that the increase in IL-1β levels may fluctuate over time and may synchronize with the worsening of symptoms; however, longitudinal studies are required to evaluate this hypothesis. Studies have shown that the positive symptoms of SCZ are mainly related to the overactivation of dopaminergic neurons in the mesolimbic system [41]. It has been shown that the number of dopaminergic neurons in the fetal brain increases after early infection or in the presence of maternal immune stimulation during pregnancy [42]. IL-1β induces the differentiation of mesencephalic-derived progenitor cells into dopaminergic neurons [43, 44] and induces continuous hippocampal inflammation, leading to severe depletion of developing neuroblasts and skewing of the fate of neural progenitor cells in the dentate gyrus of the hippocampus [45]. *IL-1β* mRNA levels have also been found to be associated with decreased Broca-area volume and verbal fluency in SCZ [46]. These studies indicated that pro-inflammatory cytokines have important effects on neurotransmitter systems, neurogenesis, and neurodevelopment in SCZ patients. Combined with these findings, our results suggest that IL-1β may be involved in pathophysiological changes leading to first-episode SCZ, and that baseline levels of IL-1β may serve as biomarkers for evaluating the severity of positive symptoms in patients with first-episode SCZ.

Tie-2, the homologous receptor for angiogenin-1 and -2 (Ang1 and Ang2), is a transmembrane tyrosine kinase receptor that is predominantly expressed in vascular endothelial cells and controls angiogenesis and vascular remodeling [47]. Aside from its vascular functions, Tie-2, particularly the Tie-2/Ang signaling axis, plays an important role in inflammatory processes. Previous studies have shown that Tie2/Ang-2 signaling induces the expression of intercellular adhesion molecule 1 and vascular cell adhesion molecule 1 and promotes leukocyte adhesion and transport to inflamed tissues in response to inflammatory cytokines [48, 49]. Macrophage Tie-2 signaling can promote a pro-inflammatory environment by inducing the production of IL-6 and the chemokine macrophage inflammatory protein 1α, while antagonizing this pathway can reduce inflammatory responses [50]. Others have previously demonstrated that Ang-2 contributes to establishing an overall immunosuppressive environment via upregulation of IL-10 by Tie-2-expressing monocytes/macrophages (TEMs) and by regulation of T cell expansion [50]. In addition, one study showed that the mRNA and protein levels of Tie-2 are decreased in lipopolysaccharide-induced inflammatory responses in mice [20]. Another study found a reduced number of TEMs in the peripheral blood circulation during an inflammatory state [20, 51]. In our study, we revealed that Tie-2 expression is reduced in patients with first-episode SCZ as compared to that in healthy controls. Tie-2 expression in vivo depends on the blood flow. Vasodilation, which occurs during inflammation in relation to the decrease in endothelial shear stress, reduces Tie-2 expression; however, the regulatory mechanism in inflammation remains to be studied [52]. Combined with the above research, our results suggest that SCZ is associated with chronic low-grade inflammation.

Clinical trials have confirmed that inflammation is causally involved in atherosclerotic events in humans. Recently, increased IL-1 activity has been associated with increased risk and severity of cardiovascular diseases [53]. Low Tie-2 receptor expression may lead to vascular complications associated with infection and inflammation [54]. Significantly, cardiovascular disease is two-fold higher in people with SCZ than in the general population and is the main reason for a shortened life expectancy and death among patients with SCZ [16, 17]. Moreover, compared with psychotic symptoms or disease duration, negative SCZ symptoms have a higher degree of impact on the increased risk of cardiovascular disease in these patients [55]. Our results showed that low Tie-2 receptor expression in the case group was significantly and positively correlated with negative symptoms. Although few clinical studies have supported the Tie-2 receptor as a diagnostic marker, various inflammatory cytokines, including TNF-α, IL-1β, and IL-11, influence Tie-2 receptor expression, making analysis of this receptor an attractive method for predicting vascular disease [52, 56]. Consistent with previous studies, our study showed a significant correlation between Tie-2 expression and IL-1β levels. Additionally, the results of the ROC analysis suggested that IL-1β and Tie-2 had high specificity and sensitivity for predicting the progression of clinical symptoms in the case group. Therefore, we speculate that patients with first-episode SCZ may have inflammation-related pathological angiogenesis, which is related to the occurrence and development of cardiovascular disease. The Tie-2 receptor may be an important predictor of increased cardiovascular risk caused by negative symptoms in patients with SCZ. Nevertheless, it remains unclear whether the changes in Tie-2 expression are the outcome of changes in cytokine levels or are caused by a vascular inflammatory response in patients with SCZ. Therefore, further research is needed to solve this problem comprehensively.

Considering the research methods related to inflammation-associated cytokine levels, as compared with the traditional methods of ELISA and Luminex assays, is worthwhile. The main advantage of the present study is that we used MSD to measure the level of inflammatory cytokines. This method is more sensitive, accurate, and precise. Our study had the following limitations: First, the cross-sectional design of this study limited our causal inferences regarding the role of inflammatory cytokines and Tie-2 in first-episode SCZ patients. Prospective longitudinal studies are required to obtain more accurate causal information on disease risk predictors in the future. Second, the sample size in the present study was relatively small; thus, future studies should target larger cohorts of SCZ patients. Third, to avoid drug interference with the research results, we recruited FEDN SCZ patients. However, it is noteworthy that antipsychotic drugs are considered to be one of the main causes of cardiovascular disease; therefore, further studies on different antipsychotics are needed to obtain additional results from a larger sample of patients with SCZ. Finally, our study did not assess confounding factors, such as age, diet, psychological stress, and lifestyle, which may affect the immune system and inflammatory response.

## Conclusions

In summary, our results showed that patients with first-episode SCZ have pro-inflammatory and vascular inflammatory responses, reflecting low-grade inflammation. Tie-2 and IL-1β may be important mediators between inflammation and vascular dysfunction in patients with SCZ and may also be one of the predictors of increased cardiovascular disease in this population. Future prospective longitudinal studies should explore the role of Tie-2 and inflammatory cytokines in the vascular inflammation process in SCZ. Interventions aimed at reducing inflammation and vascular risk may help to reduce vascular endothelial dysfunction and, in turn, prevent cardiovascular morbidity and mortality in SCZ.

## Data Availability

Considering the protection of patient privacy, the data used in this manuscript will not be disclosed to the public, but according to reasonable requirements, the dataset are available from the corresponding author.

## Abbreviations

AUC: Area under the curve
Ang1/2: Angiogenin-1/2
BPRS: Brief Psychiatric Rating Scale
BMI: Body mass index
DSM-5: Diagnostic and Statistical Manual of Mental Disorders-5
ELISA: Enzyme-linked immunosorbent assay kit
FEDN: First-episode drug-naïve
IL: Interleukin
IQR: Interquartile range
MINI: Mini-International Neuropsychiatric Interview
MSD: Meso Scale Discovery electrochemiluminescence assay system
PANSS: Positive and Negative Syndrome Scale
ROC: Receiver operating characteristics
SCZ: Schizophrenia
TNF-α: Tumor necrosis factor-α
Tie-2: Tyrosine kinase receptor with immunoglobulin and epidermal growth factor homology domain 2
TEMs: Tie-2 expressing monocytes/macrophages
